# The relationship between austerity and food insecurity in the UK: A systematic review

**DOI:** 10.1016/j.eclinm.2021.100781

**Published:** 2021-03-15

**Authors:** Rosemary H. Jenkins, Shirin Aliabadi, Eszter P. Vamos, David Taylor-Robinson, Sophie Wickham, Christopher Millett, Anthony A. Laverty

**Affiliations:** a Public Health Policy Evaluation Unit, Department of Primary Care and Public Health, School of Public Health, Imperial College London, Charing Cross Campus; The Reynolds Building; St Dunstan's Road; London W6 8RP, UK; b Global Digital Health Unit, Department of Primary Care and Public Health, Imperial College London, London, UK; c Department of Public Health, Policy and Systems, Institute of Population Health, University of Liverpool, Liverpool, UK

**Keywords:** Austerity, Welfare reform, Food insecurity, Foodbank, Food aid, Public health, Health inequalities

## Abstract

**Background:**

In 2010, the UK government implemented austerity measures, involving reductions to public spending and welfare reform. We aimed to systematically review the relationship of austerity policies with food insecurity including foodbank use in the UK.

**Methods:**

We undertook a narrative systematic review (CRD42020164508) and searched seven databases, grey literature, and reference lists through September 2020. Studies with austerity policies (including welfare reform) as exposure and food insecurity (including foodbank use as a proxy) as study outcome were included. We included quantitative longitudinal and cross-sectional studies. Two reviewers assessed eligibility, extracted data directly from studies, and undertook quality assessment.

**Findings:**

Eight studies were included: two individual-level studies totalling 4129 participants and six ecological studies. All suggested a relationship between austerity and increased food insecurity. Two studies found that austerity policies were associated with increased food insecurity in European countries including the UK. Six studies found that the welfare reform aspect of UK austerity policies was associated with increased food insecurity and foodbank use. Sanctions involving delays to benefits as a response to a claimant not actively seeking work may increase food insecurity, with studies finding that increases of 100 sanctions per 100,000 people may have led to increases of between 2 and 36 food parcels per 100,000 population.

**Interpretation:**

UK austerity policies were consistently linked to food insecurity and foodbank use. Policymakers should consider impacts of austerity on food insecurity when considering how to reduce budget deficits.

**Funding:**

NIHR School for Public Health Research.

**Research in Context Panel**

**Evidence before this study**

Previous studies suggest that austerity policies may increase food insecurity, but evidence on this has yet to be synthesised. Prior to the present study, we searched MEDLINE; Embase; PsycINFO; Health Management Information Consortium (HMIC); Business Source Ultimate; CINAHL; and Web of Science databases on July 3rd, 2019 using terms including “austerity”, “public sector spending”, or “welfare reform” and “food insecurity”, “food poverty”, or “hunger” to determine if a systematic review assessing the relationship between austerity policies and food insecurity had been undertaken. We found no reviews on this topic; thus there is a need for a systematic review on austerity policies and food insecurity.

**Added value of this study**

To our knowledge, we are the first to systematically review quantitative studies of the relationship between austerity policies and food insecurity and foodbank use in the UK. Two studies found that implementation of austerity policies was associated with food insecurity in European countries including the UK. The remaining six studies found that welfare reform as part of UK austerity was associated with increased food insecurity and foodbank use.

**Implications of all the available evidence**

We recommend that policy makers consider potential impacts of austerity measures on food insecurity, particularly welfare reform which leads to reductions in incomes of the poorest in society. We recommend that such approaches are not used by governments to reduce budget deficits, and that the UK government considers removing features which cause benefit reductions and delays as these can considerably reduce individuals’ abilities to afford food. Further quantitative investigation of the relationship between austerity policies and food insecurity is suggested, particularly regarding aspects other than welfare reform such as changes in public sector spending, and this research would be facilitated by routine measurement of food insecurity.

Alt-text: Unlabelled box

## 1 Introduction

Many European countries including the UK implemented austerity measures after the 2008 Great Recession [1,2]. Austerity is defined as “official actions taken by the government, during a period of adverse economic conditions, to reduce its budget deficit using a combination of spending cuts or tax rises” [3]. In the UK, austerity policies led to prominent changes in public sector spending. This involved wide-ranging but heterogeneous decreases in funding of local authorities – local authority spending was reduced by 23.4% in real terms between 2009–10 and 2014–2015 [4]. More deprived areas and cities experienced greater cuts in funding [4], [5], [6]. Impacts differed between authorities and for different services, for example, planning and development services generally experienced greater cuts than environmental services [7].

Another aspect of UK austerity policies was changes to the benefits system and welfare reform. New policies included a benefit cap to limit the amount households could receive and removal of child benefit if the household contained a higher rate tax payer [8,9]. Some policies directly decreased benefits or led to decreases through conditionality, such as changes to the way that housing benefits (Local Housing Allowance) were calculated which meant that low income private renters received less in housing benefit, sanctions for failing to meet criteria for active job-seeking such as searching for jobs, and increases in the amount of hours worked to qualify for working tax credit [8,10,11]. There were also changes to eligibility, including reassessments for benefits leading to more stringent tests and higher levels of conditionality, for example, disability benefits, previously the Disability Living Allowance, changed to a new system, Personal Independence Payments, which involved reassessment for benefits against different criteria [8,12,13]. Additionally, there was the introduction of the two child policy, restricting the child elements of benefits to the first two children [14]. Furthermore, a new benefits system, Universal Credit, was designed to unify legacy benefits to lead to one monthly salary payment and absorbed the reductions, caps, and changes in eligibility previously mentioned [15]. Analysis by the Equality and Human Rights Commission found that the tax and welfare reforms announced since 2010 were regressive, with a greater impact on those with lower incomes and vulnerable groups, especially people with disabilities, lone parents, certain ethnic groups, and children [16].

Food insecurity is defined by the Food and Agriculture Organisation (FAO) as “limited access to food… due to lack of money or other resources” [17]. Food insecurity can range from mild food insecurity (worrying about being able to obtain food), moderate food insecurity (compromising quality and variety of food, reducing quantities, skipping meals) to severe food insecurity (experiencing hunger) [17]. It may lead to decreased fruit, vegetable, and protein consumption, increased processed food consumption, disordered eating patterns and lower levels of vitamins and minerals [18], [19], [20], [21]. Food insecurity is also associated with poorer physical health, higher body weight and obesity (especially in adult women), and chronic disease, irrespective of knowledge about how to eat healthily [22], [23], [24], [25]. Food insecure children are also significantly more likely to have poorer health and behavioural problems [26], [27], [28]. Food insecurity is also associated with wider social issues including problems with housing and substance abuse [29].

In 2018, the UK was estimated to have the highest level of food insecurity in Europe [30], and some previous studies have suggested that UK austerity policies may be linked to food insecurity [8,31,32]. Welfare reform may have led to decreases in household incomes and difficulties affording food [8,13]. A descriptive study of foodbank users in County Durham found that high proportions of them had been affected by welfare reform [32]. Additionally, changes in public sector spending may affect services that provide a safety net, such as social care and advice services, which could contribute to these issues [31]. A regressive relationship between austerity policies and food insecurity would widen nutrition and health inequalities. However, no previous studies have synthesised quantitative research on the association between austerity policies and food insecurity in the UK. Thus, we aimed to systematically review the relationship between austerity policies and food insecurity and foodbank use in the UK.

## 2 Methods

### 2.1 Search strategy and selection criteria

We undertook a systematic review following a protocol registered on the International Prospective Register of Systematic Reviews (CRD42020164508). Studies’ inclusion criteria were as follows:

1) Population: UK based (without restrictions on time period).
2) Exposure: The exposure measure must include austerity policies or welfare reform as a component of austerity policies in the UK; different facets of welfare reform such as sanctions and removal of the spare room subsidy were considered as exposure to austerity policies.
3) Comparison: Those not exposed to austerity. Geographical variation in austerity measures was also used to enable comparisons between exposures and outcomes.
4) Outcome: Food insecurity was defined as the outcome, and we accepted all definitions and scales as outcomes, including foodbank use as a proxy.
5) Longitudinal studies were included, as were cross-sectional studies if they exploited geographical variation in austerity measures.
6) Studies were included if their methods were empirical and they quantitatively tested the relationship between austerity policies and food insecurity.

Studies were excluded if:

1) They were qualitative or descriptive studies.
2) They were conference abstracts.
3) They were not in English (due to the UK focus of the study).

Food insecurity can be measured in different ways, such as the Food Insecurity Experience Scale (FIES) Survey Module which consists of eight questions regarding access to food such as running out of money or worrying about that, eating less, skipping meals, or losing weight because of being unable to buy food [33]. Another measure used is the ability to afford eating a meal with meat, chicken, or fish (or vegetarian equivalent) every second day [34]. We also included concepts of food deprivation, food hardship, and food poverty. Usage of foodbanks and food assistance can be used as a proxy as it correctly identifies an individual or household experiencing food insecurity, although may underestimate food insecurity as only a proportion of food insecure households will access a foodbank [35]. We therefore also included papers which used data on foodbanks opening and usage, and use of other food assistance as a proxy for food insecurity.

We searched seven databases in September 2020: Business Source Ultimate; CINAHL; Embase; Health Management Information Consortium (HMIC); MEDLINE; PsycINFO; and Web of Science. We used search terms including “austerity”, “public expenditure”, “benefit cut”, and “public sector financing” and “food insecurity”, “food deprivation”, “food hardship”, and “foodbank”. For example, our search of Embase was as follows: “austerity OR public expenditure OR benefit claim OR benefit cut* OR spending cut* OR welfare reform OR sanctio* OR social protection OR (spending adj3 change*) OR public spending OR public sector financing OR public sector spending OR universal credit OR local authorit* OR public financ* OR budget cut* OR welfare regime OR welfare state OR debt crisis OR budget deficit* OR troika OR Eurozone crisis OR economic adjustment program* AND malnutrition OR food insecurity OR food insecurity [MeSH term] OR food security OR food security [MeSH term] OR food deprivation OR food deprivation [MeSH term] OR nutrition security OR food hardship OR food poverty OR food insufficiency OR food bank* OR foodbank OR food assistance OR food assistance [MeSH term] OR food charity OR food pantr* OR food distribution OR food parcel* OR soup kitchen* OR community kitchen* OR community food program*, limit to English”.

We also searched for relevant grey literature. We manually searched all publications on the websites of organisations undertaking research on government expenditure and policies or poverty and inequalities: the Institute for Fiscal Studies, the Joseph Rowntree Foundation, and Oxfam. We also manually searched the websites of The Food and Agriculture Organisation, The Trussell Trust, FareShare, the Food Foundation, and the Independent Food Aid Network due to their research into and proximity to issues relating to food insecurity and foodbank use. We also searched OpenGrey Europe database and the UK government's publications database, and also manually examined each individual submission to the evidence submission call for the visit to the UK by Professor Philip Alston, United Nations Special Rapporteur on extreme poverty and human rights in 2018, which were available on the UN Office of the United Nations High Commissioner for Human Rights website. Finally, we searched references of included papers. References were imported into Covidence software for screening [36]. We screened studies in line with PRISMA guidance [37], with RJ and SA screening the title and abstracts and then full texts of studies independently. Disagreements were discussed and resolved between RJ and SA.

### 2.2 Data analysis

Data were extracted into a data extraction form independently by RJ and SA, and discrepancies discussed and resolved. We extracted author, year and title; ethics and funding; location; time points; participants’ characteristics; details regarding data collection and study design; number of participants; exposure and outcome assessments; statistical methodology including covariates; and key findings including Odds Ratios and β coefficients. We extracted data directly from studies; additional data were not sought from authors although authors were contacted if methodological clarifications were required. RJ and SA independently assessed study quality using the Newcastle Ottawa Scale and discussed and resolved conflicting scores [38,39]. This method uses the following criteria to assign a score out of nine: selection (representativeness of sample, sample size, exposure method – maximum 4 points), comparability (maximum 2 points) and outcome (ascertainment of the method and statistical test – maximum 3 points). We considered a score of 0–4 to be low quality, 5–6 to be medium quality and 7–9 to be high quality. We performed a narrative analysis of study findings, assessing outcome measures and exposures, as the heterogeneity of studies did not allow for quantitative assessment or meta-analysis. However, we calculated the change in number of food parcels for an increase of 100 sanctions per 100,000 adults where studies used different denominators to enable comparison of rates. We used harvest plots to display and summarise study results, with each bar representing a study, the y axis representing study quality according to the Newcastle Ottawa Scale and the x axis denoting the effect direction [40]. We structured the review by outcome in terms of food insecurity and foodbank use.

### 2.3 Role of the funding source

The funders had no role in the study design, data collection, data analysis, data interpretation, or the writing of the report. The views expressed are those of the authors and not necessarily those of the funders. The corresponding author had full access to all data in the study and had final responsibility for the decision to submit for publication.

## 3 Results

**Fig. 1:**
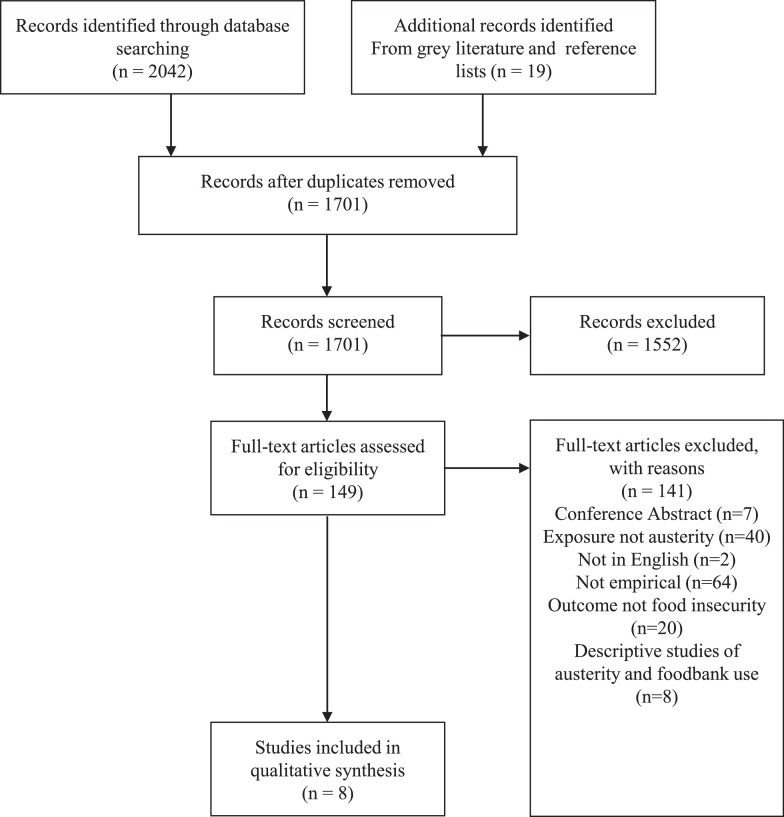
PRISMA Flow Diagram detailing screening and selection of studies.

We identified 2042 studies through database searching, 360 of which were duplicates – all 1682 non-duplicate studies were screened by title and abstract (see Fig. 1). We then screened the full text of 131 studies. We excluded 123 studies - seven conference abstracts, 38 papers without austerity as the exposure (generally concerning patterns of food insecurity or the impact of the Great Recession without following austerity measures), two papers not in English, 55 papers that were not empirical, 20 papers where the outcome was not food insecurity or relating to foodbank usage and one study which was only descriptive with regard to exposure to austerity policies and foodbank use. Full text screening identified seven studies and one study from reference lists eligible for inclusion in the review. No studies were found in the grey literature search – we screened the full text of 18 relevant grey studies, but all were excluded. Two studies did not have austerity as the exposure, nine were not empirical, and seven did not quantitatively test the relationship between austerity and food insecurity and foodbank use. Thus, eight studies in total were included in this review and are summarised in Table 1 [41], [42], [43], [44], [45], [46], [47], [48].

**Table 1:** Detailed Description of Studies included in this review.

Author & Year	Location	N	Exposure	Years	Methods & Outcome Measures	Outcome category and key findings	Quality^a^
Loopstra 2015a [42]	European countries including UK	Not stated	Commencement of austerity	2005–2013	Time trend analysis ‘Can I just check whether your household could afford a meal with meat, chicken, fish or vegetarian equivalent every second day if you wanted it?’ Outcome: Eurostat data **(food insecurity)**	**Food Insecurity:** Since 2010, the prevalence of food insecurity was about 2.71 (95% CI: 0.56, 4.85) percentage points greater than expected based on previous trends. This corresponds to an excess of 13.5 million people (95% CI: 2.8, 24.2) living with food insecurity.	Low
Loopstra 2015b [43]	UK	Not stated (375 local authorities)	Sanctions, cuts in central government or local authority welfare spending	2006–2007, 2007–2008, 2008–2009, 2009–2010. 2010–2011, 2011–2012, 2012–2013, 2013–2014	Logistic regression with lagged variable approach for foodbank opening, adjusted for the unemployment, Gross Value added (local economic conditions) and proportion of people identifying as Christian. Linear regression used for food parcel distribution, adjusting for the local authority's capacity to provide food (accounting for number of foodbanks and years of foodbank operations). OR of foodbank opening and percentage point change in foodbank use. Outcome: Data from The Trussell Trust **(foodbank use)**	Each one percentage point higher rate of adverse sanction decisions per claimant on foodbank opening: one year later: 1.08 (95% CI: 0.95, 1.22); on foodbank use: 0.09 (95% CI: 0.01,0.17) *p* < 0.05 *Increase of 100 sanctions per 100,000 adults associated with an increase of 1.95 food parcels per 100,000 adults (calculated by authors) (2010–2013).* Each 1% cut in central government welfare spending on foodbank opening: one year later: OR: 1.16 (95% CI: 0.95, 1.41), two years later: OR: 1.59 (95% CI: 1.25, 2.03) *p* < 0.001; on foodbank use: β: 0.16 (95% CI: 0.10, 0.22) *p* < 0.001. Each 1% cut in local authority spending on foodbank opening: one year later: 1.07 (95% CI: 1.03, 1.11) *p* < 0.001, two years later: 1.06 (95% CI: 1.02, 1.11) *p* < 0.001; on foodbank use: −0.021 (95% CI: −0.05, 0.01)	Medium
Loopstra 2018 [41]	UK	424 food banks in 259 local authorities	Sanctions	2012–2013, 2013–2014, 2014–2015, 2015–2016	Fixed effects model for rate of sanctions in local authority population and number of food parcels distributed. First difference model for increasing sanctions and number of Job Seekers Allowance Claimants from previous quarter and adult foodbank usage. Model 1: no linear and quadratic time trends; Model 2: adjusting for the scale of foodbank operations and foodbank operating time, Model 3: adjusting for linear and quadratic time trends including dummy variables for seasons and first quarter a foodbank operated, and local authority fixed effects. Outcome: Data from The Trussell Trust. **(foodbank use)**	Relationship between sanctions applied/ JSA claimants and number of adult foodbank users in local authorities with foodbanks, 2012–2015: Per 10 additional sanctions per 100,000 adults: Model 1) 6.44 (SE: 0.87) <0.001 Model 2) 6.35 (SE: 0.87) *p* < 0.001 Model 3) 3.36 (SE: 0.84) *p* < 0.001 *Increase of 100 sanctions per 100,000 adults associated with an increase of 33.6 food parcels per 100,000 adults (calculated by authors) (2012–2016).* Per 10 additional JSA claimants per 100,000 adults: Model 1) −1.81 (SE: 0.20) *p* < 0.001; Model 2) −1.73 (SE: 0.20) *p* < 0.001; Model 3) −0.76 (SE: 0.24) *p* < 0.01 Dynamic relationship between the change in number of sanctions applied/ JSA claimants from quarter-to-quarter and change in numbers using foodbanks: Per 10 additional sanctions applied from previous quarter: Model 1) 5.20 (SE: 1.12) *p* < 0.001 Per 10 fewer sanctions applied from previous quarter: Model 1) n/a; Model 2) −1.79 (SE: 0.73) *p* < 0.05 Per 10 additional Job Seekers Allowance claimants from previous quarter: Model 1) 0.11 (SE: 0.28); Model 2) −0.038 (SE: 0.28)	High
MacLeod 2018 [44]	UK (Glasgow)	3614	Being impacted by welfare reforms (binary) as a self-reported exposure	2015	Logistic regression with covariates: gender, age, household structure, longstanding illness of disability, mental health problems, employment status, citizenship status, experience of life events, food affordability difficulties and fuel affordability difficulties. Outcome: **Foodbank use** User vs. non-user (did not need to use a foodbank) vs. non-accessor (did not use a foodbank because they did not want to or because they were unable to access one). Glasgow Community Health and Wellbeing Study (GoWell).	Proportion of respondents impacted by welfare reforms by foodbank user group (n in brackets): Under-occupation deduction – user: 13.6%, non-user: 3.6%, non-accessor: 6.3%, chi-squared: 36.0 *p* < 0.01 Personal Independence Payment/ Disability Living Allowance changes – user: 15.7%, non-user: 3.3%, non-accessor: 12.8%, chi-squared: 77.7 *p* < 0.01 Employment and Support Allowance changes – user: 18.8%, non-user: 3.0%, non-accessor: 11.3%, chi-squared: 104.5 *p* < 0.01 Housing benefit changes – user: 18.0%, non-user: 4.0%, non-accessor: 17.9%, chi-squared: 138.2 *p* < 0.01 Working tax credit changes – user: 7.2%, non-user: 3.4%, non-accessor: 6.6%, chi-squared: 8.5 *p* < 0.05 Sanctions – user: 19.0%, non-user: 2.8%, non-accessor: 22.1%, chi-squared: 190.4 *p* < 0.01 Odds ratios of reporting use of foodbanks Impacted by welfare reforms, OR: 2.293 (95% CI: 1.459, 3.604) *p* < 0.05	Medium
Prayago 2018 [45]	UK (London Boroughs of Islington, Wandsworth, Lambeth)	515 (270 from foodbank, 245 from Advice Centre)	Not receiving benefits due to sanction or delay. Also comparison of characteristics of foodbank users vs. advice centres	Apr – Aug 2016	Number and proportions of foodbank users compared to advice centre users (**foodbank use)**. Pooled regression analysis for changes in household food security score (step 1: gender, age, education attainment, employment status and benefits entitlement; step 2: financial strain, adverse life events). Outcome: 10-item Household Food Security Model which assesses food insecurity over the past 12 months and categorises as high food security, marginal, low, or very low food insecurity (**food insecurity)**	**Foodbanks:** high proportion of foodbank users affected by welfare reform (higher than advice centres). Benefit entitlements Yes, Foodbanks n: 175 (64.8%) *p* < 0.01, Advice Centres n: 157 (64.1%); No—due to sanction or delay, Foodbanks n: 57 (21.1%), Advice Centres n: 8 (12.3%); Formerly receiving, Foodbanks n: 8 (17.4%), Advice Centres n: 38 (15.5%); Never received, Foodbanks n: 30 (11.0%), Advice Centres n: 42 (17.1%) **Food Insecurity:** Association of benefit entitlement with food insecurity, β value and 95% CI): Benefits entitlement (never received = ref) Currently receiving benefits 0.41 (95% CI: −0.33, 1.08) Not receiving due to sanction or delay 1.01 (95% CI: 0.02, 1.97), *p* < 0.05 Formerly receiving benefits 0.117 (95% CI: −1.02, 1.21)	Medium
Reeves 2017 [46]	21 countries in Europe including UK	Not stated (21 countries and 166 country-years)	Austerity defined as a government reducing expenditure in any two years between 2008 and 2012 (Y/N)	2004–2012	First difference regression models with covariates: percentage change in consumer prices for food minus the percentage change in wages, GDP, unemployment rate, year and type of welfare regime of country. Outcome: ‘Can I just check whether your household could afford a meal with meat, chicken or fish every second day if you wanted it?’ Eurostat Data **(food insecurity)**	Announced and begun implementing austerity (Yes = 1) β 0.636 (95% CI: −0..006, 1...293) (percentage point change in food deprivation)	Medium
Reeves 2020 [48]	UK	76,734 observations from 2656 postcode districts (ie. postcode-district months)	Number of households receiving UC in each postcode district available	August 2015 – December 2017	Linear regression with covariates: proportion of working age population receiving Job Seekers Allowance or claiming Universal Credit for unemployment at the Local Authority level, linear time trends, and seasonality. Also used a Granger causality test and multi-level approaches to examine different aspects of the relationship. The multi-level model clustered at Job Centre Office and local authority levels while adjusting for number of foodbank distribution centres in postcode district and an interaction term between proportion of households on Universal Credit and number of food bank distribution centres in postcode district. Outcome: Food parcel distribution. Trussell Trust data (**foodbank use**).	Modelling association between change in proportion of households receiving universal credit over time and change in proportion of households receiving food parcels: 1 percentage-point increase in proportion of households on Universal Credit: 0.011 (SE: 0.0018) *p* < 0.01 1 percentage-point increase in the proportion of households on Universal Credit in the previous month: 0.011 (SE: 0.0019) *p* < 0.01 1 percentage-point increase in the proportion of households on Universal Credit in the previous month (including lagged measure of food parcel distribution – in effect Granger Causality Test): 0.086 (SE: 0.0016) *p* < 0.01 Multilevel model enabling clustering at job centre level and local authority level: 1 percentage-point increase in the proportion of households claiming Universal Credit in month prior: 0.0085 (SE: 0.0024) *p* < 0.01 Per additional 1 percentage-point more households claiming from the month prior: 0.012 (SE: 0.00061) *p* < 0.01	High
Sosenko 2017 [47]	UK	1130 from main foodbank users survey, 206 for referral agency survey, 28 for foodbank manager survey	Sanctions, failed Personal Independence Payment assessments, removal of the spare room subsidy	Oct-Nov 2018 (foodbank user survey), April-May 2019 (manager survey)	Fixed effects regression controlling for number of foodbanks, real weekly value of out-of-work benefits, number of work seekers per 1000 working age population, percent of working age benefit claimants on Universal Credit, number of people on health-related benefits per 1000 working age population. Outcome: Data from The Trussell Trust (**foodbank use)**	Number of Job Seekers Allowance /Employment and Support Allowance sanctions per 1000 working age population: β: 0.31 (SE: 0.10, 95% CI: 0.11–0.50), *p* = 0.002. *Increase of 100 sanctions per 100,000 adults associated with an increase of 31 food parcels per 100,000 adults (calculated by authors) (2011/12–2018/19).* Number of failed Personal Independence Payment assessments per 1000 working age population β: 0.93, (SE: 0.37, 95% CI: 0.21, 1.65), *p* = 0.012, Number of households subject to the removal of the spare room subsidy per 1000 working age population: β: 0.68 (SE: 0.13, 95% CI: 0.41, 0.94) *p* < 0.001. 94% of people referred to foodbanks classified as food insecure. 80% of food insecure households classified as severely food insecure.	Medium

a Assessed using the Newcastle Ottawa Scale.

Two studies were at the individual level with a combined sample size of 4129 [44,45], and the remaining studies were ecological [[41], [42], [43],[46], [47], [48]]. Five were longitudinal [[41], [42], [43],^46^,^48^] and three were cross-sectional [44,45,47]. One study used the commencement of austerity in time as the exposure, with another defining countries as having introduced austerity if they had governments that reduced expenditure in any two years between 2008 and 2012 [42,48]. The remaining studies used welfare reform as their exposure, including benefit delays or sanctions, introduction of Universal Credit, and the removal of the spare room subsidy policy [41,[43], [44], [45],^47^,^48^]. Three studies used food insecurity [42,45,46] and five had foodbank use as the outcome [41,43,44,47,48]. Only one of these quantified the severity of food insecurity rather than prevalence [45]. Two studies were high quality [41,48], five were medium quality [43], [44], [45], [46], [47], and one was low quality [42]. Our quality assessment included consideration of statistical tests and confounders, in line with the Newcastle Ottawa Scale [38,39]. Generally studies used regression models or t-tests [41,[43], [44], [45], [46],^48^]. A variety of covariates were adjusted for, including individual characteristics: age, gender, household income, longstanding illness, and ability to afford food or fuel [[43], [44], [45],^47^]. Some studies using foodbank data adjusted for foodbank operating characteristics including opening times, as well as local level factors such as proportion of ethnic minorities and unemployment rates [41,43,47]. National level studies included GDP and unemployment to adjust for the 2008 recession, or compared actual trends with existing trends [42,46]. Five studies were by the same author [[41], [42], [43],^46^,^48^]. Four studies used data from the same data source – The Trussell Trust, a large foodbank network in the UK set up as a Christian charity which collates data on foodbank use into a large, national dataset [41,43,47,48].

**Fig. 2:**
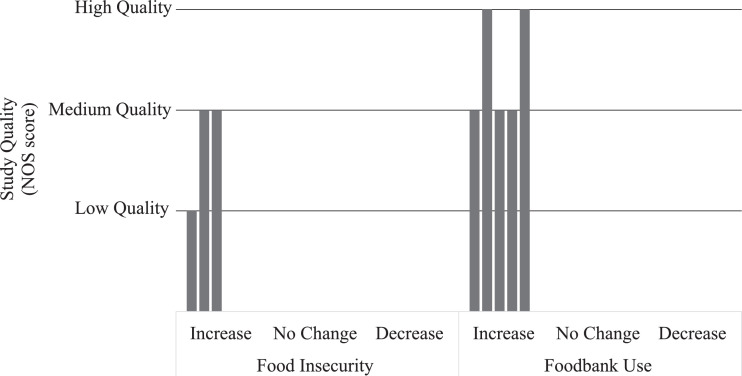
Harvest plot of the association between austerity and food insecurity and foodbank use. Each bar represents a single study, with the height of the bar representing study quality via the Newcastle Ottawa Scale. The x axis indicates effect direction, for food insecurity and foodbank use separately.

Three studies used food insecurity measures as the outcome [43,45,46]. Two were located across European countries including the UK and were concerned with European austerity measures after the 2008 recession [42,46]. In one study, a country was considered to have implemented austerity policies if they had reduced government expenditure in any two years between 2008 and 2012; thus the UK was considered to be one such country [46]. The authors concluded that implementing austerity policies had an independent effect on food insecurity (β: 0.64, (95% CI: −0.01, 1.29)) that was independent of the gap between wages and food prices, with the outcome measure as being able to afford a meal with meat, chicken, fish, or vegetarian equivalent every second day, from the Eurostat database [46]. Another study using the same outcome measure found that the prevalence of food insecurity was 2.71 percentage points (95% CI: 0.56, 4.85) higher than expected based on previous trends and coinciding with austerity beginning in Europe [42]. The third study investigated foodbank users compared to advice centre users in the London boroughs of Islington, Wandsworth, and Lambeth and found that not receiving benefits due to sanctions or delays was associated with an increase in severity of food insecurity (β: 1.01 (95% CI: 0.02, 1.97) [45]. This study was the only one to measure severity of food insecurity using a scale with multiple categories (high, marginal, low or very low food security). Thus, all three studies found a potential association between austerity policies and an increase in food insecurity, as shown in Fig. 2.

Five studies investigated the welfare reform aspect of the UK government's austerity policies [41,43,44,47,48]. Different measures were used as outcomes, including number of referrals to foodbanks, number of children or adults served by foodbanks, and number of parcels distributed. A study investigating food insecurity of foodbank users found that 94% of people referred to a foodbank were classified as food insecure [47], which validates the use of this measure as a proxy. All found that welfare reform was associated with foodbank use [41,43,44,47,48]. One study found that being impacted by welfare reform (compared to not being impacted) was associated with doubled odds of using foodbanks (OR: 2.29 (95% CI: 1.46, 3.60) *p* < 0.05) [44]. Each 1% cut in central government welfare spending was significantly associated with higher odds of foodbanks opening two years later (OR: 1.59 (95% CI: 1.25, 2.03) *p* < 0.001) and foodbank use (β: 0.16 (95% CI: 0.10, 0.22) *p* < 0.001); a similar pattern was seen for each 1% decrease in local authority welfare spending on odds of foodbanks opening (OR: 1.06 (95% CI:1.02, 1.11) *p* < 0.001), but the relationship with foodbank use was not statistically significant (β: - 0.021 (95% CI: - 0.05, 0.01)) [43]. Three studies reported that an increase in sanctioning was associated with an increase in food parcels distributed by foodbanks [41,43,47]. One found that an increase of 100 sanctions per 100,000 population was associated with an increase of 31 food parcels per 100,000 population, while another study found a similar increase of 34 food parcels per 100,000 population [41,47]. An increase of 100 sanctions per 100,000 adults was linked to an increase of 2 food parcels per 100,000 in the third study [43]. One hundred failed Personal Independence Payment assessments (a UK benefit for those with long-term disabilities or ill-health which had more stringent criteria than previous disability benefit) per 100,000 was associated with an additional 93 food parcels per 100,000 population being distributed per year [47]. Additionally, an increase of 100 people subject to the removal of the spare room subsidy led to 68 additional food parcels per 100,000 people [47]. Not receiving benefits due to sanctions or delays was more common in foodbanks (21% of users) compared to advice centre users (12% of users) [45]. An increase in proportion of households on Universal Credit was also consistently and significantly associated with an increase in households receiving food parcels using a number of different statistical methods (β: 0.09 (*p* < 0.01)) [48].

## 4 Discussion

This systematic review has collated all available quantitative evidence on the relationship between austerity policies and food insecurity in the UK. Studies consistently found that austerity policies were associated with food insecurity and foodbank use in the UK. Two studies found that austerity policies were associated with an increase in food insecurity; welfare reform including cuts in welfare spending, sanctions, and removal of the spare room subsidy were also associated with food insecurity and foodbank use in six studies.

Our review has reported that welfare reform was associated with foodbank use. Sanctioning, disability benefit reassessments, the removal of the spare room subsidy and Universal Credit were all significantly associated with an increase in foodbank use. This is in line with studies which suggest that these changes can lead to an inability to afford food, destitution, and foodbank use [49], [50], [51]. Local area studies of foodbanks suggest that high proportions of foodbank users do so due to benefit changes and delays. Percentages of people using foodbanks who have experienced benefit changes (including changing to a different benefit or benefits being stopped entirely) or delays (including sanctions) ranged from 21% of foodbank users in Islington, Wandsworth, and Lambeth [45] to 54% of foodbank users in County Durham [32]. These assessments of reasons for foodbank use support the findings of our systematic review that welfare reform may play a role in foodbank use. Additionally, we have also described one study which found that an increase in the proportion of households on Universal Credit was associated with an increase in households receiving food parcels [48]. This potential impact of Universal Credit is supported by studies identifying increases in foodbank use following Universal Credit rollout [52,53]. While Universal Credit was designed to unify the complex legacy benefits system, features such as the five week wait, two child policy limit, and sanctions may lead to increases in food insecurity and foodbank use as people switch over to the new system [15,54,55].

We have also reviewed the limited evidence on the impact of austerity policies on food insecurity in the UK. Two studies conducted in European countries including the UK concluded that austerity policies were associated with an increase in food insecurity, and that this was independent of wages and food prices [42,46]. However, the 95% confidence interval of one of these studies did include the possibility of no impact, and the studies did not assess mechanisms behind potential impacts of austerity policies or differences between countries [46]. While welfare reform may have affected food insecurity, impacts of other aspects of UK austerity policies such as changes in public sector spending have not been examined. Investigating effects of such aspects is important as they may have independent effects on food insecurity due to the depth and heterogeneity of public sector spending changes [5], [6], [7], and also as potential impacts of public sector spending and welfare reform may be linked. For example, welfare reform coupled with reductions in local authority funding for support services and public transport may mean that already food insecure individuals may struggle to access foodbanks and advice centres [56]. Furthermore, only one study investigating the impacts of welfare reform used food insecurity as the outcome, in contrast with the higher volume of studies on foodbank use, and this was the only study to measure severity of food insecurity [45]. Further research in this area may have been hindered by a lack of comprehensive measurement of food insecurity in the UK. A positive development is that the UK has recently introduced a single, nationwide measure for food insecurity, which will be reported in 2021 [57]. Our review highlights the importance of this as well as the importance of quantifying severity of food insecurity as well as prevalence [58].

Our review supports existing evidence which suggests that welfare reform and an unravelling safety net have led to increasingly insecure lives with difficulty affording food for the poorest members of UK society [59]. Reductions in incomes due to austerity policies have been experienced almost exclusively in the lowest 50% of incomes, who have experienced a reduction in income of approximately 10% due to the changes in benefits and Universal Credit announced since 2010 [16]. These reductions in resources have also coincided with increases in food prices, which mean that households in the lowest income decile would need to spend 74% of their disposable income to eat healthily [60]. Income insufficiency is associated with food insecurity and an inability to afford a healthy diet, and thus a reduction in income for those with already low resources may increase food insecurity [61]. Food insecurity may lead to unhealthy diets and disordered patterns of eating [18,21]. Thus, welfare reform may have significant regressive impacts on nutrition and health for both adults and children [62], and may have widened already existing inequalities in nutrition and health.

This review has several strengths. We took a broad view regarding outcome measures including using foodbank use as a proxy for food insecurity. We searched seven databases, relevant grey literature, and reference lists to ensure this review was comprehensive. The scope of our review in the UK enabled a specific and comprehensive overview of the impact of UK austerity policies. This review also has some limitations. All studies consistently reported that austerity policies were associated with increased food insecurity regardless of study quality, and thus we cannot rule out the risk of publication bias in these studies. Furthermore, half of studies included in this review use The Trussell Trust data as outcome data, which may be a non-representative subset of the food insecure population, particularly as The Trussell Trust does not oversee all foodbanks in the UK and some food insecure individuals will attend different foodbanks or not attend foodbanks [35]. Thus, these findings are likely to underestimate the scale of the impact of austerity in food insecurity. Additionally, disentangling impacts of austerity from impacts of recessions is important, as the studies generally did not control for the economic recession [63]. However, the majority of impacts we describe were either associated with specific austerity policy changes such as benefit sanctions, had a considerable lag time to the 2008 recession, or examined actual trends compared with existing trends. Finally, other work has explicitly examined the severity of austerity policies and the severity of their impacts, but we were unable to quantify this for food insecurity due to the studies included in this review.

In conclusion, our review suggests that austerity policies have had a direct and consistent negative impact on food insecurity in the UK. It highlights the need for robust and routine measurement of food insecurity, which would facilitate further and more robust research in this area. While we have synthesised evidence on the role of welfare reform in food insecurity, there is a gap in the literature regarding the role of other mechanisms of austerity – for example through changes in public expenditure. Research ascertaining impacts on severity as well as prevalence of food insecurity would also be beneficial. Impacts of austerity measures on individuals not on benefits and not directly impacted by welfare reform needs to be further elucidated. Overall, our systematic review highlights the need for further research into the impacts of UK austerity policies, particularly in the areas of nutrition and health. A strong safety net providing adequate resources for people to maintain healthy diets will be an important part of mitigating health impacts of the COVID-19 pandemic. Recent controversies in the UK over free school meals and holiday hunger serve as a reminder of the intersection of existing inequalities and COVID-19 impacts. Therefore, we recommend that policy-makers consider the potential impacts of austerity measures, particularly in terms of welfare reform leading to benefit caps and reductions, on food insecurity as the UK government tries to reduce budget deficits caused by the COVID-19 pandemic. We recommend that policy-makers do not introduce fiscal consolidation measures which further erode the safety net for the poorest in society as a response to budget deficits.. We would also recommend that UK policy-makers consider how to re-establish the safety net for the most vulnerable in society and remove features which cause benefit reductions and delays, as these can drastically reduce individuals’ resources and affect their ability to afford food.

## Data sharing statement

The data used in this study were extracted from publicly available studies and are available from the corresponding author upon reasonable request.

## Contributors

RJ, AL, and EV conceptualised and designed the study. RJ and SA did the literature search, study selection, and quality assessment. RJ and SA undertook data extraction. All authors contributed to the interpretation of the findings. RJ wrote the first draft of the manuscript. All authors contributed to manuscript writing and revisions and approved the final version.

## Declaration of Competing Interest

The authors have nothing to disclose.
